# Vaginal Cytokine Profiles Associated with High-Risk Human Papillomavirus, Sexually Transmitted Microbe Co-Detection, and Cervical Lesions

**DOI:** 10.3390/microorganisms14102243

**Published:** 2026-10-03

**Authors:** Habtamu Biazin Kebede, Dareskedar Tsehay, Selamawit Mekuria, Ola Forslund, Christer Borgfeldt, Mats Jerkeman, Tamrat Abebe, Adane Mihret

**Affiliations:** 1 Department of Microbiology, Immunology and Parasitology, College of Health Sciences, Addis Ababa University, Addis Ababa 1165, Ethiopia; habtamu.biazin@aau.edu.et (H.B.K.); tamrat.abebe@aau.edu.et (T.A.); 2 Armauer Hansen Research Institute, Addis Ababa 1165, Ethiopia; 3 Department of Medical Oncology, Lund University, 893 17 Lund, Sweden; 4 Department of Translational Medicine, Lund University, 680 40 Malmö, Sweden; 5 Department of Obstetrics and Gynecology in Linköping, Department of Biomedical and Clinical Sciences, Linköping University, SE-58185, 580 02 Linköping, Sweden

**Keywords:** cervical lesion, cytokines, high-risk HPV, sexually transmitted microbes, co-detection, vaginal swabs

## Abstract

Persistent hrHPV infection is the necessary cause of cervical cancer, but local immune responses and sexually transmitted infections or genital mycoplasma may influence viral persistence and lesion progression. This study examined vaginal cytokine profiles according to hrHPV and sexually transmitted microbe (STM) detection and assessed their association with cervical lesions. Self-collected vaginal samples were obtained from 80 women classified into four mutually exclusive groups: hrHPV infection alone (n = 16), STM detection alone (n = 16), hrHPV/STM co-detections (n = 26), and negative controls (n = 22). Among 80 participants, 10 (13%) were women living with HIV. Detection of target organisms was performed using real-time PCR on a CFX96 Real-Time PCR Detection System (Bio-Rad, Hercules, CA, USA). Histological analysis included 33 women: 25 were women with normal histology or cervical intraepithelial neoplasia 1 (CIN1), and 8 women with CIN2+ lesions. Vaginal cytokine concentrations (pg/mL) were analyzed using the Invitrogen ProcartaPlex™ Multiplex Immunoassay (Vienna, Austria). Statistical analyses assessed group differences in cytokine levels. Women with hrHPV/STM showed significantly elevated levels of Eotaxin (*p* = 0.04), IL-2 (*p* = 0.045), IL-10 (*p* = 0.02), and IP-10 (*p* = 0.04) compared to negative controls. Elevated IL-2 (*p* = 0.016) was also observed in women with STMs alone; women with hrHPV infection showed lower IL-15 levels (*p* = 0.02). Higher concentrations of TNF-α (*p* = 0.04), IL-1β (*p* = 0.02), and EGF (*p* = 0.01) were associated with CIN2+ lesions. Women with hrHPV/sexually transmitted microbe co-detection showed higher levels of Eotaxin, IL-2, IL-10, and IP-10, and the association of hrHPV alone with lower IL-15 levels. The findings also show that higher levels of EGF, IL-1β, and TNF-α were associated with CIN2+ lesions.

## 1. Introduction

Persistent hrHPV infection is the necessary cause of cervical cancer, yet only a proportion of infected women develop high-grade cervical lesions [1]. Local immune responses, including alterations in cytokine and chemokine production, may contribute to inter-individual variation in cervical disease progression. Previous studies have reported altered peripheral cytokine profiles in women with hrHPV infection and cervical lesions, including differences in IL-10, IFN-γ, IL-1β, and TNF-α [2]. However, findings have varied according to the population studied, the biological specimen analyzed, and the stage of cervical disease.

STIs may also influence the local immune response. *Chlamydia trachomatis*, *Neisseria gonorrhoeae*, and *Trichomonas vaginalis* have been associated with changes in inflammatory responses, yet the vaginal cytokine profiles in women with concurrent hrHPV and STIs are poorly characterized [3,4]. The host immune system and these infections have a complex and heterogeneous interaction. Comprehending the mechanisms that underlie them is crucial for formulating tailored preventative and treatment measures [5].

Previous studies reported that *Mycoplasma genitalium* (MG), *Mycoplasma hominis* (MH), *Ureaplasma urealyticum* (UU), and *Ureaplasma parvum* (UP) produce different types of immune responses by producing varying levels of pro- or anti-inflammatory cytokines [6,7]. MG and UU are primarily associated with a greater production of pro-inflammatory cytokines such as IL-1β and IL-8 [8]. In contrast, MH is only associated with increased production of IL-1β. UU and UP have also been associated with inflammatory responses [9] involving cytokines, including TNF-α and IL-6 [7,9], although the strength and clinical relevance of these associations remain uncertain.

An effective response to infections depends on the balance and regulation of cytokines. Thus, cytokine profiling in biological specimens provides insights into the host’s inflammatory milieu and immune status [10]. Individual differences in the cytokine milieu can be associated with a variety of factors, including the type and the location of the disease, co-infections, microbiome, hormonal status, and age-related factors [11].

This process is especially important for STIs and high-risk HPV (hrHPV) infections because both local and systemic immune responses may have a significant association with the infection and clinical outcomes of these infectious diseases [12]. Persistent hrHPV infection is the necessary cause of cervical cancer; however, progression to high-grade lesions and invasive cancer is multifactorial, with the local vaginal immune environment representing one of several contributing factors. Sexually transmitted infections may influence this by altering immune responses [13].

Women with hrHPV infection and cervical cancer cases exhibited altered specific cytokine profiles [14]. Research has demonstrated an association between hrHPV infection and altered cytokine profiles, including reduced Interferon gamma (IFN-γ), increased Interleukin-10 (IL-10), and elevated levels of other immunosuppressive cytokines. This immunosuppressive environment may help promote hrHPV infection, cervical dysplasia, and malignancy [14,15].

The appropriate response to sexually transmitted infections is typically demonstrated by a careful balancing act between pro- and anti-inflammatory cytokines. Increased levels of cytokines like TNF-α, IL-1, and IL-6 indicate bacterial pathogen infections [16], such as those brought on by *Neisseria gonorrhoeae* and *Chlamydia trachomatis*, almost always result in a strong pro-inflammatory response [12]. These cytokines are critical in initiating and sustaining the immune response, aiding in the pathogen’s clearance. Conversely, excessive inflammation can be associated with tissue damage and chronic problems such as infertility and pelvic inflammatory diseases [17,18].

This study assessed differences in vaginal cytokine profiles among sexually active Ethiopian women according to detection status, including hrHPV alone, STMs alone, hrHPV/STM co-detection, and negative controls. The study also examined the association of cytokines with cervical lesions.

**Research in Context.**

**Evidence before this study:** Persistent high-risk human papillomavirus (hrHPV) infection is the necessary cause of cervical cancer. Not all hrHPV-positive women will progress to high-grade cervical lesions. Thus, other factors, such as the local immune environment, may influence whether an infection leads to cervical disease. Sexually transmitted infections (STIs) such as *Chlamydia trachomatis*, *Neisseria gonorrhoea*, and *Trichomonas vaginalis* may modulate local immune responses and influence hrHPV persistence. Previous studies suggested that genital mycoplasmas such as *Mycoplasma genitalium* (MG), *Mycoplasma hominis* (MH), *Ureaplasma urealyticum* (UU), and *Ureaplasma parvum* (UP) produce different types of immune responses by producing varying levels of pro- or anti-inflammatory cytokines. However, limited evidence is available on the association of these microorganisms’ detection or hrHPV/sexually transmitted microbes (STMs) co-detection and cervical lesion severity with vaginal cytokine profiles. **Added value of this study:** This study provides new information about the vaginal immune environment in women with hrHPV infection, STMs, and hrHPV/STM co-detections. We measured cytokines in self-collected vaginal samples and demonstrated distinct cytokine patterns according to infection status and cervical lesions. Participants with hrHPV/STM had higher levels of Eotaxin, IL-2, IL-10, and IP-10. Women infected with hrHPV had lower levels of IL-15. Epidermal growth factor (EGF), Interleukin-1β (IL-1β), and Tumor Necrosis Factor-α (TNF-α) levels were higher in women with CIN2+ lesions than in women with normal histology or CIN1. **Implications of all the available evidence:** Our results indicated that the detection of hrHPV co-detection with STMs is associated with alterations of the local immune milieu in hrHPV-infected women. The study therefore established an association between certain vaginal cytokine profiles and cervical histological abnormalities. Self-sampled vaginal measurements may have the potential to detect non-invasive high-grade cervical lesions, but this requires confirmation in larger prospective studies. In low-resource settings, these findings may support further investigation of non-invasive approaches to cervical lesion risk stratification. Their clinical value should be evaluated in larger prospective studies. 

## 2. Methods and Materials

### 2.1. Study Setting and Study Population

This cross-sectional analysis included 80 women of reproductive age who underwent HPV-based cervical cancer screening. Participants were tested for hrHPV, three sexually transmitted infections (STIs), and four genital mycoplasmas. 

### 2.2. Sample Collection and Laboratory Procedures

**Sample collection:** Vaginal samples were collected and transported using Copan eNAT^®®^ tubes containing 2 mL of liquid medium and nylon-flocked swabs (Copan Italia S.p.A., Brescia, Italy). Samples were collected between November 2021 and June 2022.

**HPV detection and typing**: HPV detection and typing were performed using AnyplexII HPV HR (Seegene Inc., Seoul, Republic of Korea), which detects and genotypes 14 hrHPV types (i.e., 16, 18, 31, 33, 35, 39, 45, 51,52, 56, 58, 59, 66, and 68) in a single reaction tube. Real-time PCR assays were performed using a CFX96 Real-Time PCR Detection System (Bio-Rad, Hercules, CA, USA), as previously described [19].

**STM detection:** STM detection was performed using the Allplex™ STI-EA real-time multiplex kit on the CFX96™ thermal cycler (Bio-Rad, CA, USA). The Multiplex RT-PCR Allplex™ STI Essential Assay detects three classical STIs, including *N. gonorrhoeae*, *C. trachomatis*, and *T. vaginalis*, and four genital mycoplasmas, including *M. genitalium*, *M. hominis*, *U. urealyticum*, and *U. parvum*, in a single tube using dual priming oligonucleotide (DPO™) and multiple detection temperatures (MuDT™), providing individual Ct values for multiple pathogens in one channel. The assay has a sensitivity and specificity of about 95% for most pathogens, with internal and external quality control procedures ensuring accuracy and reproducibility [20].

As directed by the manufacturer, the Allplex™ STI-EA detection kit uses 5 μL of template DNA and 15 μL of master mix in a total volume of 20 μL. Then, real-time PCR was performed using Allplex™ STI essential assay kits on the CFX96 Bio-Rad Real-Time thermocycler (Bio-Rad, CA, USA) in association with the IVD Seegene software, V5.1.

The real-time PCR was carried out as per the manufacturer’s instructions. The thermal cycle conditions consisted of an initial incubation at 50 °C for 4 min to activate the UDG system and prevent contamination, and pre-denaturation at 95 °C for 15 min, followed by 50 cycles of alternating incubations: 95 °C for 30 s, 60 °C for 1 min, and 72 °C for 30 s. An internal control targeting human beta-globin and positive controls were run in each run [21].

**HIV status:** HIV status was obtained from existing clinical records to be included as a potential confounding and risk factor variable [22].

**Histology:** Cervical biopsies were collected from women who tested positive for hrHPV. The biopsy specimens were processed, stained, and reviewed by pathologists. Histological findings were classified based on the CIN system of classification, as previously described [19,23].

**Negative controls:** The negative-control group included women who tested negative for hrHPV, STIs, genital mycoplasmas, and HIV infection.

**HrHPV/STM co-detection:** In this study, the term co-detection refers to the co-occurrence of hrHPV and at least one other sexually transmitted infection, including *Chlamydia trachomatis* (CT), *Neisseria gonorrhoeae* (NG), *Trichomonas vaginalis* (TV), or genital mycoplasmas, including *Mycoplasma genitalium* (MG), *Mycoplasma hominis* (MH), *Ureaplasma urealyticum* (UU), and *Ureaplasma parvum* (UP). This definition applies regardless of the number of HPV genotypes detected or the number of STMs detected. Therefore, co-detected does not refer to multiple hrHPV types alone, multiple STMs alone, or all possible combinations, but rather to the coexistence of hrHPV with other sexually transmitted infections and genital mycoplasmas, together referred to as sexually transmitted microbes (STM(s)).

**Determination of Cytokine Concentration**: Concentrations of 45 cytokines, chemokines, and growth factors were measured using the ProcartaPlex™ Multiplex Immunoassay kit (Invitrogen, Vienna, Austria), a magnetic bead-based multiplex platform that enables simultaneous quantitative detection of multiple analytes from a single sample.

The panel included BDNF (Brain-Derived Neurotrophic Factor), EGF (epidermal growth factor), Eotaxin (CCL11), FGF-2 (Fibroblast Growth Factor 2, also called basic FGF), GM-CSF (Granulocyte-Macrophage Colony-Stimulating Factor), GRO-α (Growth-Regulated Oncogene-alpha, also CXCL1), HGF (Hepatocyte Growth Factor), IFN-α (Interferon alpha), IFN-γ (Interferon gamma), IL-1RA (Interleukin-17 Receptor Antagonist), IL-1α, IL-1β, IL-2, IL-4, IL-5, IL-6, IL-7, IL-8, IL-9, IL-10, IL-12p70, IL-13, IL-15, IL-17A, IL-18, IL-21, IL-22, IL-23, IL-27, IL-31, IP-10 (Interferon Gamma-Induced Protein 10/CXCL10), LIF (Leukemia Inhibitory Factor), MCP-1 (Monocyte Chemoattractant Protein-1/CCL2), MIP-1α (Macrophage Inflammatory Protein-1 alpha/CCL3), MIP-1β (Macrophage Inflammatory Protein-1 beta/CCL4), NGF-β (Nerve Growth Factor beta), RANTES (Regulated on Activation, Normal T Cell Expressed and Secreted/CCL5), PDGF-BB (Platelet-Derived Growth Factor BB), PIGF-1 (Placental Growth Factor 1), SCF (Stem Cell Factor/c-Kit ligand), SDF-1α (Stromal Cell-Derived Factor 1 alpha/CXCL12), TNF-α (Tumor Necrosis Factor alpha), TNF-β (Tumor Necrosis Factor beta/Lymphotoxin-alpha), VEGF-A (Vascular Endothelial Growth Factor A), and VEGF-D.

**Normalization and standard curve:** The concentration of the samples was calculated by plotting the expected concentration of the standards against the median fluorescent intensity (MFI) generated by each standard. A 5-parameter logistic (5-PL) standard curve was used to calculate cytokine concentrations.

The result was analyzed according to the operation manual for the Luminex™ instrument. Normalization and the standard curve were determined after exporting the run data in .csv format and navigating to the ProcartaPlex™ Analysis App on the ThermoFisher Connect website: https://apps.thermofisher.com/apps/procartaplex accessed on 20 July 2025.

Cytokine concentrations were quantified using a ProcartaPlex 45-plex immunoassay (Thermo Fisher Scientific, Vienna, Austria). The lower limit of detection (LLOD) for each analyte was derived from the mean signal of blank samples + 3 × SD (n = 2). Intra-assay CVs were calculated from replicates across single runs, and CVs < 20% were considered acceptable, except at near-LLOD levels, where higher variability is expected.

Cytokine concentrations above the lower limit of detection (LLOD) but below the lower limit of quantification (LLOQ) were included in the analysis using the observed values provided by the assay. These values were considered detectable but semi-quantitative and were analyzed using non-parametric statistical methods (Kruskal–Wallis and Mann–Whitney U tests). However, samples below the LLOD were flagged as ‘OOR < [value]’ and excluded from downstream analysis. Standard curves exhibited linearity (R^2^ > 0.98) for all targets. Measurements below the LLOD were set to the lowest standard value; values above the LLOD were set to the highest standard concentration [24].

### 2.3. Statistical Analysis

Descriptive and inferential analysis was carried out using GraphPad Prism v10.3 (GraphPad Software Inc., San Diego, CA, USA). The results were reported as medians and interquartile ranges (IQRs) or means with standard errors for descriptive statistics. Group analysis and median (IQR) were reported only if ≥50% of samples detected a given cytokine, chemokine, and growth factor.

The different cytokine levels were compared with the non-parametric Kruskal–Wallis’s test. The Kruskal–Wallis’s test was also used to compare cytokine levels across multiple groups defined by infection status, including hrHPV alone, STI alone, hrHPV/STM co-detection, negative control groups, and HIV-negative, HIV-positive, and HIV/HPV co-infected groups. Multiple cytokine analyses were performed simultaneously; therefore, we used the false discovery rate (FDR) to minimize the possibility of false positive results in these experiments. The FDR was chosen as an alternative to a *p*-value threshold because it considers the total number of statistical comparisons that are being made. An overall comparison across all tested cytokines was performed on each cytokine separately with the non-parametric Kruskal–Wallis test. Then, the *p*-values that resulted from these comparisons across all analyzed cytokines were corrected for FDR using the Benjamini–Hochberg method. If there was a statistically significant difference in groups as compared to the reference group, then post hoc pairwise comparisons were completed using Dunn’s test, with Holm adjustments made for the number of pairwise comparisons.

The Mann–Whitney U tests were used to compare differences between the two histological grades (normal cervical histology/CIN1 and CIN2+) and HIV-positive and HIV-negative groups. The *p*-value of each Mann–Whitney U test was adjusted using the Benjamini–Hochberg false-discovery-rate method for multiple testing of cytokines measured. If an FDR-adjusted *p*-value is less than 0.05, it is determined to be statistically significant.

All detectable cytokine levels underwent log transformation to ensure raw values were homogeneous and normal for β-coefficients and multiple linear regression analysis. Then, multiple linear regression was performed to evaluate the relationships between hrHPV, STMs, HIV, age, and log10-transformed cytokine concentrations. The models were adjusted for relevant co-detection and potential confounders. Multicollinearity was assessed using the variance inflation factor (VIF); a VIF less than 5 was acceptable. All comparisons were two-sided, and a 0.05 level of significance was used. 

## 3. Results

### 3.1. Sociodemographic and Clinical Characteristics of the Study Population

The median age of the participants was 32, with a range of 24–44 years. All the participants were married and had one sexual partner in their lifetime, and no reported tobacco or alcohol use. None of the participants reported active symptoms of lower vaginal tract infections at the time of sample collection.

Based on microbial detection status, participants were classified into four mutually exclusive groups: HrHPV infection alone, STM detection alone, HrHPV/STM co-detection, and negative control. Of the participants, 13% (10/80) of the women lived with HIV. Out of 42 women with hrHPV, 33 (78.6%) had histology results (Table 1).

### 3.2. Cytokine Expression Level

Out of the 45 cytokines analyzed, 42 (93%) were expressed above the detection limit in one or more samples. Eighteen cytokines (40%) were expressed above the lower limit of detection (LLOD) in more than half of the vaginal swabs, including epidermal growth factor (EGF), Eotaxin, HGF, Interleukin-17 Receptor Antagonist (IL-1RA), Interleukin 2 (IL-2), Interleukin-4 (IL-4), Interleukin-10 (IL-10), Interleukin-12 p70 (IL-12p70), IL-13, IL-15, IL-17A (CTLA-8), IL-27, Interferon Gamma-Induced Protein 10 (IP-10, (CXCL10)), LIF, Monocyte Chemoattractant Protein 1 (MCP-1 (CCL2)), Stem Cell Factor (SCF), TNF alpha, and Vascular Endothelial Growth Factor A (VEGF-A).

Seven (16%) cytokines, including Eotaxin, Hepatocyte Growth Factor (HGF), Tumor Necrosis Factor alpha (TNF-α), Leukemia Inhibitory Factor (LIF), Interleukin-12p70 (IL-12p70), IL-17 Receptor Antagonist (IL-1RA), and IL-4, were expressed in all the vaginal samples (100%). Placental Growth Factor 1 (PIGF-1), TNF-beta, and VEGF-D expression were below the lower limit of detection (LLOD) at any level in both the infected and control groups (Table 2).

### 3.3. Cytokine Concentration in hrHPV, Sexually Transmitted Microbes, hrHPV/Sexually Transmitted Microbe Co-Detection, and Controls

Women with hrHPV/STM co-detection had higher concentrations of EGF, Eotaxin (CCL11), IL-2, IL-4, IL-10, IP-10, and VEGF-A compared with controls. Eotaxin, HGF, TNF alpha, LIF, IL-12p70, IL-1RA, IL-4, and IL-10 were detected in all the groups analyzed (Table 3).

**Figure 1:**
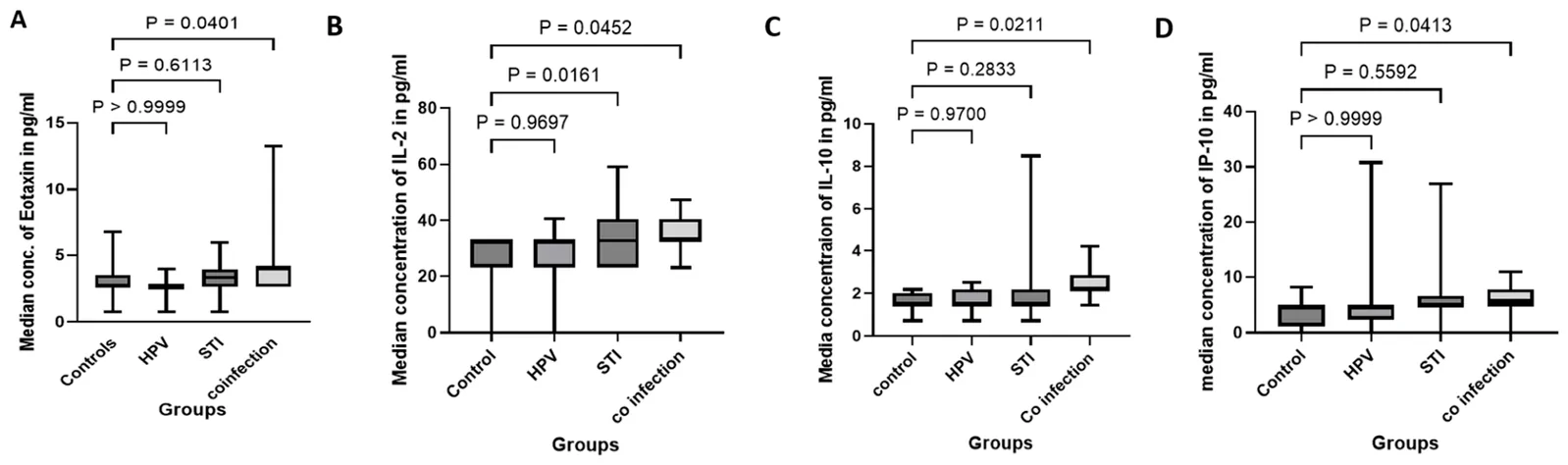
The median vaginal cytokine concentrations in participants with hrHPV, STMs, and hrHPV/STM co-detection in comparison to negative controls (**A**–**D**). Note: Statistical differences were determined by the Kruskal–Wallis’s test, and Dunn’s post hoc test with Holm adjustment was used for pairwise comparisons.

In women with hrHPV/STM co-detection, we found significantly higher median Eotaxin concentration compared to controls (*p* = 0.040) (Figure 1A). Median IL-2 concentrations were significantly higher in the STM alone (*p* = 0.016) and hrHPV/STM co-detected (*p* = 0.045) compared to negative controls (Figure 1B). The median concentration levels of IP-10 (*p* = 0.041) and IL-10 (*p* = 0.021) differed significantly in co-detected women compared to controls (Figure 1C,D). No statistically significant differences were observed for the remaining analytes across the four groups (Supplementary Figure S1E–T).

### 3.4. Cytokine Concentration in Normal Cervical Histology/CIN1 and CIN2+ (Table 4)

Median vaginal sample concentration levels of Eotaxin, IL-1RA, IL-2, IL-4, IL-10, IP-10, LIF, MCP-1, and VEGF were higher in the CIN2+ groups than in the normal cervical histology/CIN1 group. The median concentrations of SCF and IL-27 were lower in the CIN2+ group compared to the NCH/CIN1 group; however, these differences were not statistically significant (*p* > 0.05 for all) (Table 4).

**Table 4:** Cytokine median concentration levels across histological grades (normal cervical histology/CIN1 (n = 25) and CIN2+ (n = 8)).

Cytokines	Median (IQR) in pg/mL ⸸		*p* Value ⸸
	Normal Cervical Histology/CIN1	CIN2+	
**EGF**	24(24–24)	47(24.25–245)	0.01 *
**Eotaxin**	2.7(2.7–4.0)	3.3(2.7–5.0)	0.14
**HGF**	19(19–31)	19(19–40)	0.90
**IL-1β**	10(10–10)	14(10–36)	0.02 *
**IL-1RA**	144(144–362)	362(144–2037)	0.99
**IL-2**	33(23–31)	37(33–41)	0.06
**IL-4**	8.4(5.8–11)	9.0(6.4–12)	0.47
**IL-10**	1.7(1.5–2.2)	2.2(1.6–2.4)	0.28
**IL-12p70**	2.5(1.9–3.4)	2.5(2.5–4.3)	0.27
**IL-13**	8.6(8.6–10)	8.6(8.6–14)	0.56
**IL-15**	3.4(3.4–6.2)	3.4(3.4–3.5)	0.56
**IL-17A**	4.9(4.9–7)	4.9(4.9–8.4)	0.55
**IL-27**	50(24–50)	37(24–73)	0.61
**IP-10**	4.8(4.8–6.6)	5.7(4.8–7.8)	0.47
**LIF**	6.8(6.8–8.5)	8.5(6.8–9.7)	0.46
**MCP-1**	6.4(6.4–6.4)	9.3(6.4–13)	0.10
**SCF**	0.97(0.66–1.3)	0.66(0.66–2.2)	0.97
**TNF-α**	20(20–26)	26(21–30)	0.04 *
**VEGF-A**	6.1(4.5–7.5)	7.5(6.1–36)	0.12

Note: NCH/CIN1 = normal cervical histology or cervical intraepithelial neoplasia 1; CIN2+ = cervical intraepithelial neoplasia 2/3 or invasive cervical cancer; * Mann–Whitney U test *p*-values were adjusted for multiple testing across the 45 cytokine analytes using the Benjamini–Hochberg false discovery rate (FDR) procedure. FDR-adjusted *p*-values < 0.05 were considered statistically significant. ⸸ *p*-values were rounded to two decimal places, and values below the LLOD were excluded from downstream analysis.

**Figure 2:**
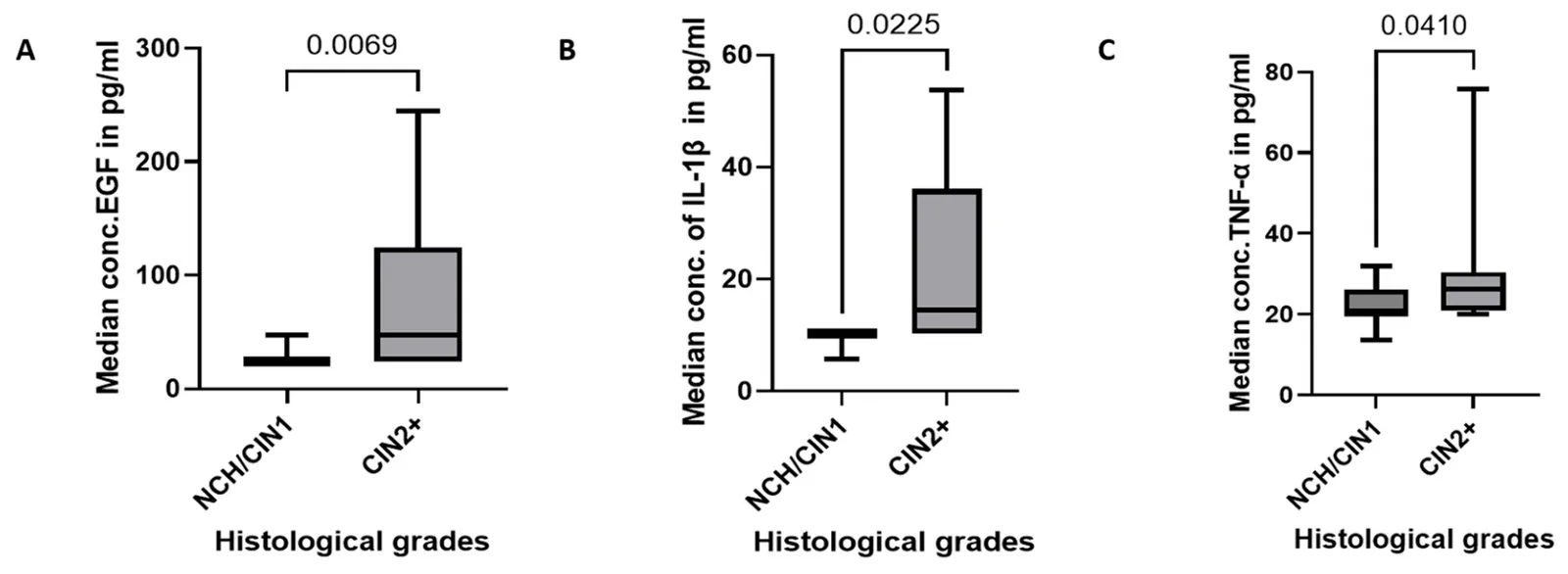
Cytokine median concentration levels of histological grades (normal cervical histology/CIN1 and CIN2+) (**A**–**C**). Note: NCH = normal cervical histology; CIN1 = cervical intraepithelial neoplasia 1; CIN2+ = cervical intraepithelial neoplasia 2/3 and invasive cervical cancer. Statistical differences were determined by the Mann–Whitney U test.

Median concentrations of EGF, IL-1β, and TNF-α differed significantly between the two histological groups (normal cervical histology/CIN1 vs. CIN2+; *p* < 0.05). Women with CIN2+ had increased median concentrations of EGF (*p* = 0.01), IL-1β (*p* = 0.02), and TNF-α (*p* = 0.04) compared with those with normal cervical histology/CIN1 (Figure 2). No statistically significant differences were found in the median concentrations of other cytokines with histological grades (*p* > 0.05) (Supplementary Figure S2D–T).

### 3.5. Cytokine Expression in Women Living with HIV and HIV/HPV Co-Detection

**Figure 3:**
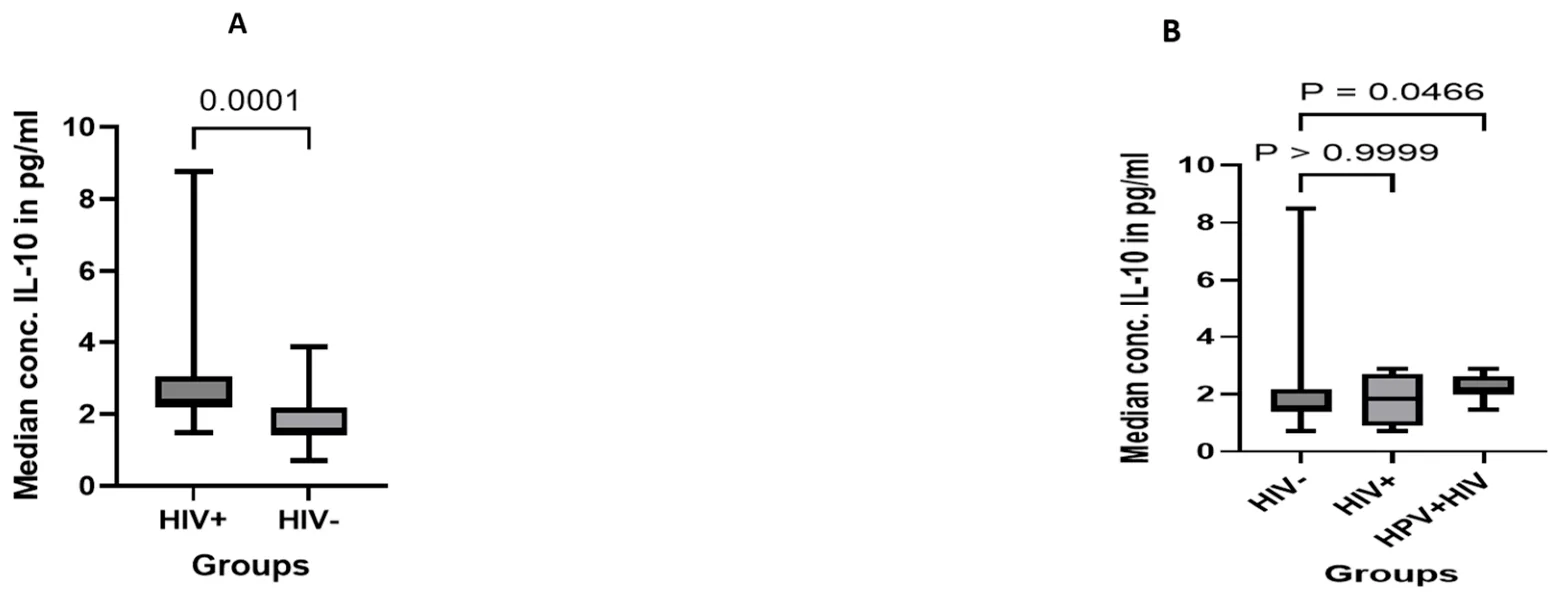
The median cytokine levels in women with HIV and HPV/HIV co-infection compared with HIV-negative women. Note: *p*-values were calculated using the Mann–Whitney U test for (**A**) and the Kruskal–Wallis’s test for (**B**), and Dunn’s post hoc test with Holm adjustment was used for pairwise comparisons.

For each of the 18 expressed cytokines, differences between women living with HIV (WLHIV) and HIV-negative women were examined. Women living with HIV (WLHIV) present a distinctive pattern of cytokines compared to HIV-negative women. WLHIV had higher levels of IL-10 than HIV-negative women (*p* < 0.0001) (Figure 3A). Vaginal swab samples from women with HIV/hrHPV co-detection demonstrated higher levels of IL-10 compared with negative controls (*p* = 0.0046) (Figure 3B). There was no observed difference in cervical cytokine expression between HIV-positive and HIV-negative women for other cytokines like EGF, Eotaxin, IL-2, IL-4, TNF-α, IP-10, IFN-γ, TGF-beta, and others (Supplementary Figure S3C–U).

In multiple linear regression analysis, none of these cytokines remained significant after adjusting for co-detection. However, hrHPV infection was positively associated with levels of log10-transformed EGF, GM-CSF, IFN-α, IFN-γ, IL-1β, IL-15, IL-17A, IL-18, IP-10, MCP-1, SCF, and VEGF-A concentration, with a positive adjusted β-coefficient.

**Figure 4:**
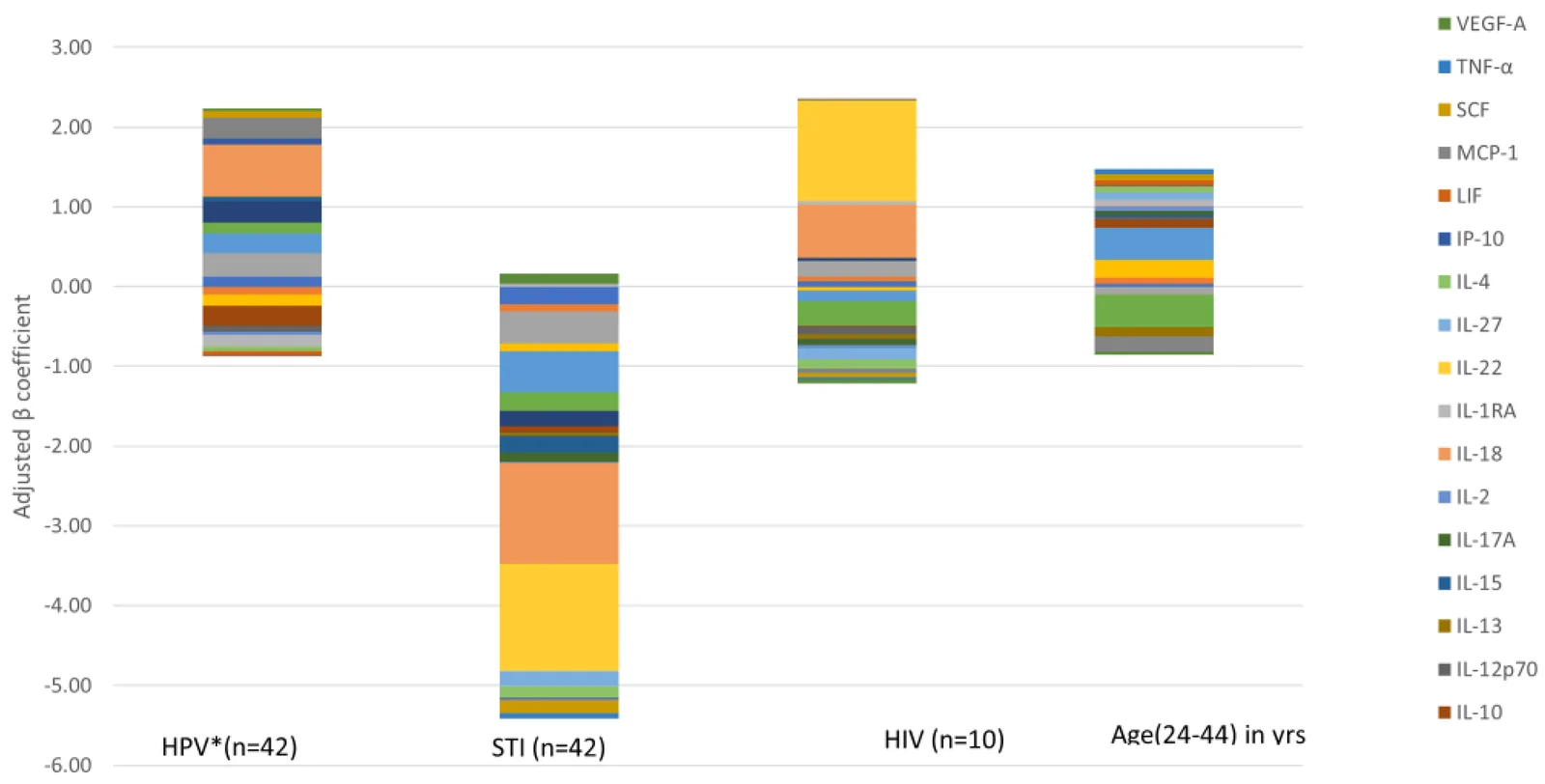
Cytokines and their relationship to demographic parameters and detection types. Cytokines were log10-transformed and standardized, and β-coefficients were computed using multiple linear regression to adjust for co-detections. * This predictor variable was analyzed independently due to the multicollinearity effect with other predictor variables, with a variance inflation factor (VIF) > 5. Points represent the adjusted β-coefficient for each cytokine, with the horizontal axis showing the direction and magnitude of the association. A β-coefficient below zero indicates lower cytokine concentration in the exposed group relative to the reference group, whereas a β-coefficient above zero indicates higher concentration.

Sexually transmitted microbe detection was also positively correlated with the log10-transformed concentration of IL-1RA and VEGF-A. Cytokine log10-transformed concentrations of LIF, IP-10, IL-22, IL-1RA, IL-18, IL-1β, GM-CSF, and Eotaxin had positive adjusted β-coefficients (Figure 4).

## 4. Discussion

Our findings reveal that women co-detected with hrHPV and STMs exhibit significantly elevated levels of pro-inflammatory and regulatory cytokines such as Eotaxin, IL-2, IL-10, and IP-10 compared to negative controls, while those with hrHPV alone show decreased IL-15 levels. Furthermore, women with cervical intraepithelial neoplasia grade 2 or worse (CIN2+) had higher concentrations of TNF-α, IL-1β, and EGF, with these cytokines associated with high-grade lesions.

In our study, hrHPV was associated with lower levels of IL-15 in the vagina samples compared to the negative controls. This finding may reflect the potential immunosuppressive nature of HPV infection. This finding is supported by reports that emphasize the immune evasion mechanisms of hrHPV infection [25]. However, this is in contrast with a study conducted in Tanzanian women infected with HPV 16/18, which reported elevated levels of IL-15 in cervical tissues [14]. IL-15 is a key cytokine involved in the activation and maintenance of natural killer (NK) cells and CD8+ T cells, both critical for controlling viral infections [26]. In hrHPV infections, the observed decrease in IL-15 may reflect the virus’s ability to evade immune surveillance, fostering an immunosuppressive microenvironment that facilitates persistent infection and the progression of histological abnormalities [14]. Previous studies suggested that reduced IL-15 levels may be associated with higher rates of HPV persistence and progression to cervical intraepithelial neoplasia (CIN) or cervical cancer. However, IL-15 levels are generally higher in cervical cancer tissues [14]. Immunological tolerance is necessary to ensure the persistence of HPV infection. Several studies have been conducted to test vaginal cytokine and chemokine levels in the past; HPV infection has had a modest systemic impact [27,28].

In our study, co-detection with HPV/STMs demonstrated a complex cytokine milieu with elevated levels of IL-2, IL-10, and IP-10. These data reflect an active, altered immune response targeted at viral clearance or lesion containment. However, comparable evidence from other populations is limited, and differences in study design, sample type, and assay methods make direct comparisons difficult. Elevated Eotaxin levels in women with sexually transmitted microbes suggest increased eosinophilic activity or inflammation in the presence of STMs [29].

In the present study, IL-2 was significantly elevated in participants with HPV/STM co-detected compared with negative controls. These findings are consistent with prior studies that have reported altered cytokine profiles, including elevated IL-2, in participants with multiple infections. Studies have shown similar immune dysregulation in co-infected patients with HPV and *Chlamydia trachomatis* [30,31], as well as in those with HIV and HPV [32], where IL-2 is often elevated, indicating a complex and potentially exaggerated immune response. This finding suggests that the immune response in co-detection may be more dysregulated, potentially contributing to the pathophysiology of co-detection. In comparison, the expression of IL-2 is typically lower in cervical cancer patients [33]. Elevated IL-2 levels, known for their role in T cell activation and proliferation, might indicate an enhanced immune response or an attempt to combat the simultaneous infections [34].

Our findings revealed that anti-inflammatory IL-10 levels in hrHPV/STM co-detection were significantly higher than in control groups, which agrees with prior studies. According to Syrjänen et al. (2009), high-grade CIN was most frequently linked to IL-10 up-regulation in tissue samples [35]. This was further supported by a study conducted on Tanzanian women infected with HPV16/18, where an elevated level of IL-10 was observed in the cervical tissue [14]. Previous research has demonstrated that Interleukin-10 (IL-10) is frequently overexpressed in cervical cancer tissues and in the blood of patients with this disease. IL-10 suppresses the immune response, potentially enabling cancer cells to evade immune detection and facilitate tumor growth. Elevated expression of Interleukin-10 is frequently associated with a shift toward a type 2 helper T cell-mediated immune response, which is generally characterized by a non-inflammatory immunological profile. This contrasts with a type 1 helper T cell-mediated response, which is more conducive to effective antitumor immunity [36,37].

Interferon-induced protein-10 (IP-10, also known as CXCL10) was elevated in hrHPV/STM co-detections compared with negative controls. This elevation is significant because IP-10 is a chemokine that plays a crucial role in immune responses. IP-10 has a strong thymus-dependent antitumor effect and primarily regulates the migration of T-cells, natural killer (NK) cells, and monocytes. IP-10, on the other hand, is crucial for controlling angiogenesis [38]. Elevated levels of IP-10 in co-detected with HPV/STMs participants suggested a heightened immune response, which could be due to the combined effects of hrHPV and STMs on the immune system. An overexpression of IP-10 is seen in infectious diseases and tumor development [38,39]. However, in advanced cervical cancer, IP-10 levels are decreased [40].

Studies indicated that WLHIV show increased levels of IL-10 [41]. Women with acute HIV infection have been found to possess significantly elevated levels of IL-10 in genital tract specimens [42]. These findings highlight the heightened inflammatory response during the early stages of HIV infection. However, a study comparing cervical cytokine expression between HIV-positive and HIV-negative women has found no significant differences in the expression levels of INF-γ, TGF-β, IL-4, and IL-12 [43]. This result is consistent with our findings. The expression levels of most of the targeted cytokines did not differ significantly in our study. This suggests that HIV infection may not uniformly alter cytokine expression in the cervical swabs.

In our study, vaginal swab samples with hrHPV/HIV co-infection had higher median IL-10 concentration levels. Previous studies have shown increased levels of IL-10 concentration in HPV/HIV co-infection compared to HIV-negative participants [44]. This suggests a predominance of Th2-type immune responses in HIV/HPV co-infected patients, potentially influencing disease outcomes.

In this cross-sectional study, EGF, IL-1β, and TNF-α were significantly elevated in women with CIN2+ compared to normal cervical histology/CIN1. Elevated EGF may drive cellular proliferation in advanced lesions, while IL-1β and TNF-α likely contribute to inflammatory and tissue remodeling processes that exacerbate disease [45,46].

Studies have shown that women with high-grade cervical intraepithelial neoplasia (CIN) or cervical cancer often exhibit altered IL-1β and TNF-α expression in the serum. This implies that in cervical cancer, local responses to inflammation triggered by HPV infection may also be reflected in systemic circulating biomarkers, which could serve as diagnostic or prognostic indicators [47,48]. Another study has indicated that cytokines such as TNF-α and IL-10 are significantly associated with hrHPV infections [49]. This result further emphasizes the role of immune dysregulation in co-infections [50,51]. This evidence collectively supports the assertion of a distinct cytokine profile in women with these infections, contributing to the pathogenesis and potential progression of cervical neoplasia [51]. Such differences correspond to previously reported variations in inflammatory mediator levels in cervical precancerous and cancerous lesions [46].

Elevated EGF contributes to enhanced cell proliferation, while IL-1β and TNF-α promote chronic inflammation and tissue remodeling, both of which are critical in advancing cervical dysplasia. Persistent HPV infection, combined with heightened inflammatory signaling, appears to accelerate the progression to CIN2+ by creating a pro-tumorigenic microenvironment [52]. Variations in cytokine concentrations across studies are likely to reflect differences in study populations, specimens, assays, and procedures. Differences in age, smoking, contraceptive use, concomitant bacterial or sexually transmitted infections, human papillomavirus (HPV) genotype, and lesion characteristics may explain inconsistencies between studies. Previously published studies report both increases and decreases in inflammatory mediators associated with HPV infection and cervical lesions [2], demonstrating the broad spectrum of variations in cytokine measurements.

Since this was a cross-sectional study, the temporal relationship between cytokine levels and CIN2+ cannot be established. The differences identified in this study do not demonstrate whether the variations in EGF, IL-1β, or TNF-α preceded or followed the development of CIN2+. Longitudinal studies are required to evaluate changes in mediators across time and to determine if the observed associations remain consistent after adjustment for relevant clinical and microbiological factors.

One of the study’s limitations is the small number of CIN2+ and individual sexually transmitted microbe cases we examined, which hinders us from drawing firm conclusions. We cannot rule out all other diseases, such as bacterial vaginosis and vulvovaginitis, as contributing factors to the immune response. Several cytokines were found to be below the baseline in more than half of the samples studied, which may have influenced the reliability and precision of the findings.

Furthermore, the analysis lacks any follow-up studies and vaginal microbiota data, which would be useful in analyzing the rates of change in the cytokines over time and the development of associated diseases and vaginal microbiome compositions.

Regarding women living with HIV and antiretroviral treatment (ART) use, this information was not uniformly available and is not included in the analysis. Inter-assay variability was not evaluated in this study. While these preliminary findings provide a compelling proof-of-concept for mucosal immunity, the small sample size of high-grade lesions (n = 8 for CIN2+) and the absence of longitudinal follow-up necessitate validation in a larger, multicenter prospective study to confirm the long-term predictive accuracy of this cytokine panel.

## 5. Conclusions

The findings revealed distinct cytokine profiles associated with hrHPV, sexually transmitted microbes (STMs), hrHPV/STM co-detections, and cervical lesions. The levels of Eotaxin, IL-2, IP-10, and IL-10 were different between controls and hrHPV/STM co-detections. EGF, IL-1β, and TNF-α were significantly elevated in women with CIN2+ compared to normal cervical histology/CIN1. These findings may indicate that some local cytokine levels might be related to infection and/or disease severity. However, due to the small sample size, the low number of CIN2+ samples assessed for histology, the cross-sectional nature of this study, and the lack of independent validation of these results, they cannot currently be used for clinical purposes. Larger prospective studies are needed to determine whether vaginal cytokine measurements can improve cervical lesion assessments. Further research is needed to assess these associations and their potential diagnostic relevance.

## Figures and Tables

**Table 1:** Characteristics of the study participants (n = 80).

Variable	Responses	Count	Percent
**Median age in years**	32		
**Age group in years**	<30	31	38.80
	≥30	49	61.20
HrHPV *	Positive	42	52.50
	HPV16	5	6.30
	HPV18	1	1.30
	HPV31	7	8.80
	HPV33	2	2.50
	HPV35	5	6.30
	HPV39	4	5.00
	HPV51	6	7.50
	HPV52	5	6.30
	HPV56	6	7.50
	HPV58	2	2.50
	HPV59	1	1.30
	HPV66	4	5.00
	HPV68	4	5.00
	Negative	38	47.50
**Histology result (n = 33)**	NCH or CIN1	25	75.80
	CIN2+	8	24.20
Sexually transmitted microbes (STMs) *	Positive	42	52.50
	NG	1	1.25
	CT	2	2.50
	TV	5	6.25
	MG	2	2.50
	MH	25	31.25
	UU	20	25.00
	UP	24	30.00
	Negative	38	47.50
**HIV serostatus**	Positive	10	12.50
	Negative	70	87.50
**Infection status of participants**	HPV alone	16	20.00
	STMs alone	16	20.00
	HrHPV/STM co-detection	26	32.50
	Negative controls	22	27.50

Note: NCH—normal cervical histology; CIN2+—cervical intraepithelial neoplasia grade 2 or worse—including CIN2—CIN3—and invasive cervical cancer; hrHPV—high-risk human papillomavirus; STMs—sexually transmitted microbes; CT—*Chlamydia trachomatis*; TV—*Trichomonas vaginalis*; MG—*Mycoplasma genitalium*; MH—*Mycoplasma hominis*; UU—*Ureaplasma urealyticum*; UP—*Ureaplasma parvum*. Negative controls tested negative for hrHPV, HIV, and the target panel of STMs. * The counts of HPV genotypes (52) and STMs (74) exceed the number of participants because multiple targets could be detected.

**Table 2:** The descriptive statistics of the targeted 45 cytokines (n = 80) and the number of samples above the LLOD (n, %).

Cytokines	Above LLOD n (%)	IQR (Q1–Q3)	Median	Mean ± SE
**BDNF**	2(3)	0.72–4.72	2.72	2.7 ± 2.0
**EGF**	52(65)	24.25–24.25	24.25	35.7 ± 4.78
**Eotaxin (CCL11)**	80(100)	2.68–3.96	2.68	3.4 ± 0.18
**FGF-2**	2(3)	13.59–50.04	31.82	31.8 ± 18.23
**GM-CSF**	20(25)	42.95–42.95	42.95	49.8 ± 4.60
**GRO alpha (CXCL1)**	2(3)	8.17–31.12	19.65	19.7 ± 11.48
**HGF**	80(100)	19.21–31.32	19.21	26.2 ± 1.76
**IFN-α**	11(14)	0.61–0.97	0.61	1.03 ± 0.25
**IFN-γ**	25(31)	3.78–3.78	3.78	4.69 ± 0.61
**IL-1α**	2(3)	0.23–0.62	0.425	0.43 ± 0.20
**IL-1β**	31(39)	10.3–18.4	10.3	14.2 ± 1.67
**IL-1RA**	68(85)	144.2–362.1	144.2	259 ± 38.98
**IL-2**	78(98)	23.0–33.69	32.66	32.2 ± 0.93
**IL-4**	80(100)	8.36–10.73	8.36	8.83 ± 0.31
**IL-5**	1(1)	-	-	-
**IL-6**	1(1)	-	-	-
**IL-7**	11(14)	1.07–2.18	1.64	1.62 ± 0.20
**IL-8 (CXCL8)**	1(1)	-	-	-
**IL-9**	1(1)	-	-	-
**IL-10**	79(99)	1.47–2.18	1.47	1.89 ± 0.12
**IL-12p70**	80(100)	2.45–3.28	2.45	2.70 ± 0.13
**IL-13**	69(86)	8.62–15.21	8.62	11.6 ± 0.92
**IL-15**	70(88)	3.39–6.19	3.39	5.69 ± 0.61
**IL-17A (CTLA-8)**	80(100)	4.94–6.96	4.94	6.14 ± 0.34
**IL-18**	6(8)	14.37–33.73	20.22	25.3 ± 6.70
IL-21 *	1(1)	-	-	-
**IL-22**	6(8)	5.21–14.63	5.21	11.5 ± 6.28
**IL-23**	1(1)	-	-	-
**IL-27**	76(95)	23.69–49.88	49.88	43.6 ± 3.19
**IL-31**	1(1)	-	-	-
**IP-10 (CXCL10)**	67(84)	4.75–6.61	4.75	6.29 ± 0.52
**LIF**	80(100)	6.76–8.47	6.76	8.11 ± 0.42
**MCP-1 (CCL2)**	44(55)	6.4–7.585	6.4	8.65 ± 1.26
**MIP-1α (CCL3)**	3(4)	1.01–3.96	2.12	2.36 ± 0.86
**MIP-1 β (CCL4)**	2(3)	10.77–56.66	33.72	33.7 ± 22.95
**NGF-β**	3(4)	1.51–3.97	2.78	2.75 ± 0.71
**PDGF-BB**	2(3)	8.08–15.02	11.55	11.6 ± 3.47
**PlGF-1**	0(0)	ND	ND	ND
**RANTES (CCL5)**	1(1)	-	-	-
**SCF**	73(91)	0.66–1.26	0.66	1.07 ± 0.10
**SDF-1α**	1(1)	-	-	-
**TNF-α**	79(99)	20.12–26.19	20.12	23.2 ± 1.0
**TNF-β**	0(0)	ND	ND	ND
**VEGF-A**	66(83)	4.52–7.53	6.06	77.4 ± 67.99
**VEGF-D**	0(0)	ND	ND	ND

Note: Interquartile range (IQR); Q1 (First Quartile); Q3 (Third Quartile); SE, standard error; unit of cytokine concentration pg./mL. Summary statistics were not calculated because only one sample was above the LLOD and was excluded from downstream analysis. ND, not detected. Summary statistics were calculated only for analytes detected above the LLOD. For analytes detected in fewer than two samples, median, IQR, and mean values were not calculated. Concentrations are reported in pg/mL.

**Table 3:** Cytokine levels in the vaginal swabs of women with hrHPV, sexually transmitted infections, hrHPV/STM co-detection, and negative controls.

Cytokine Classification	Cytokines	Median (IQR) (pg/mL) ⸸			
		Groups			
		Controls	hrHPV	STMs	hrHPV/STMs
**Chemokines**	MCP-1	6.4(6.4–6.4)	6.4(4.4–33.4)	6.4(6.4–8.6)	6.4(6.4–9.0)
	*p* value	-	>0.99	>0.99	>0.99
	IP-10	4.8(4.8–5.5)	4.8(2.4–4.8)	4.8(4.8–6.6)	5.7(4.8–7.8)
	*p* value	-	>0.99	>0.99	0.04 *
	Eotaxin	2.7(2.7–3)	2.7(2.7–3)	3.9(2.7–5.0)	4.0(4.0–5.0)
	*p* value	-	>0.99	0.61	0.04 *
**Growth Factors**	HGF	19.2(19.2–31.3)	19.2(19.2–31.3)	19.2(19.2–42.9)	19.2(31.3–31.3)
	*p* value	-	>0.99	>0.99	>0.99
	EGF	24.3(24.3–39.1)	24.3(24.3–24.3)	24.3(24.3–41.4)	24.3(24.3–47.2)
	*p* value	-	0.79	>0.99	>0.99
	SCF	0.7(0.7–1.26)	0.7(0.7–1.1)	1.3(0.7–1.3)	1.3(0.7–1.5)
	*p* value	-	>0.99	0.33	0.056
	VEGF-A	6.1(4.1–7.5)	6.1(0.0–6.1)	4.5(2.9–6.8)	6.1(2.9–9.3)
	*p* value	-	>0.99	>0.99	0.50
**Anti-Inflammatory or Immunoregulatory Cytokines**	IL-10	1.5(1.5–2.2)	1.5(1.5–1.8)	1.5(1.5–2.0)	2.2(1.5–2.9)
	*p* value	-	0.93	>0.99	0.02 *
	IL-1RA	144(144–144)	144(144.2–199)	144(144–144.2)	144(306–362.1)
	*p* value	-	>0.99	>0.99	0.25
**T-Cell Differentiation Cytokines**	IL-2	33(23–33)	33(23–33)	33(23–41)	33(33–41)
	*p* value	-	>0.99	0.02 *	0.01 *
	IL-4	8.4(8.4–9)	8.4(6–11)	8.4(7.4–10.7)	8.4(8.4–13)
	*p* value	-	0.94	0.99	0.56
	IL-13	8.6(8.6–14)	8.6(8.6–10.3)	8.6(8.6–15.2)	8.6(8.6–15.2)
	*p* value	-	>0.99	>0.99	>0.99
	IL-1RA	4.9(4.9–5.5)	4.9(3.3–4.9)	6.0(4.9–8.4)	5.5(4.9–7.4)
	*p* value	-	0.73	0.19	0.11
**Pro-Inflammatory Cytokines**	TNF-α	20.1(18.6–26.2)	20.1(13.5–25.5)	20.1(20.1–26.2)	26.2(20.1–27.6)
	*p* value	-	0.99	0.99	0.08
**Other ** **Cytokines**	IL-12p70	2.5(2.5–2.5)	2.5(1.5–2.5)	2.5(2.5–3.4)	2.5(2.5–3.6)
	*p* value	-	0.83	>0.99	0.24
	IL-15	6.2(3.4–6.2)	3.4(3.4–3.4)	3.4(3.4–8.7)	6.2(3.4–6.2)
	*p* value	-	0.02 *	>0.99	>0.99
	IL-27	23.7(23.7–49.9)	23.7(23.7–43.3)	23.7(49.9–49.9)	23.7(49.9–55.6)
	*p* value	-	0.83	>0.99	0.22
	LIF	6.8(6.8–8.5)	6.8(5.1–8.5)	6.8(5.4–8.5)	8.5(6.8–10)
		-	>0.99	>0.99	0.46

Notes: Statistical differences were determined by the Kruskal–Wallis test followed by Dunn’s post hoc test with Holm adjustment for pairwise comparisons; * Kruskal–Wallis *p*-values were adjusted for multiple testing across the cytokine analytes using the Benjamini–Hochberg false discovery rate (FDR) procedure. FDR-adjusted *p*-values <0.05 were considered statistically significant. For cytokines with a significant overall group difference, Dunn’s post hoc pairwise comparisons were performed, with *p*-values adjusted using the Holm method. ⸸ Values below the LLOD were excluded from downstream analysis.

## Data Availability

The data that support the findings of this study are available from the corresponding author upon reasonable request.

## Supplementary Materials

The following supporting information can be downloaded at https://www.mdpi.com/article/10.3390/microorganisms14102243/s1. Figure S1E–T: Distribution of vaginal cytokine concentrations across study groups (*p*-value ≥ 0.05). Box-and-whisker plots show the distribution of vaginal cytokine concentrations in study groups according to infection status. Cytokine concentrations are expressed in pg/mL. *p* values indicate comparisons against controls. Figure S2D–T: Distribution of vaginal cytokine concentrations across histological grades (*p*-value ≥ 0.05). Box-and-whisker plots show the distribution of vaginal cytokine concentrations in normal cervical histology/CIN1 and CIN2+ participants. Cytokine concentrations are expressed in pg/mL. *p* values indicate comparisons between the two histological groups. Figure S3C–U: Comparison of vaginal cytokine concentrations between women with and without HIV infection (*p*-value ≥ 0.05). Box-and-whisker plots show the distribution of selected vaginal cytokine concentrations in HIV-positive and HIV-negative participants. Cytokine concentrations are expressed in pg/mL. *p* values indicate comparisons between the two HIV serostatus groups.

## Abbreviations

5PL	Five-parameter logistic algorithm
CIN1	Cervical intraepithelial neoplasia 1
CIN2+	Cervical intraepithelial neoplasia 2/3, or cervical cancer
HrHPV	High-risk human papillomavirus
HrHPV/STM	High-risk human papillomavirus and sexually transmitted microbe co-detection
ICC	Invasive cervical cancer
LLOD	Lower limit of detection
PCR	Polymerase chain reaction
PID	Pelvic inflammatory disease
STMs	Sexually transmitted microbes
WLHIV	Women living with HIV
